# Addressing the Inflammatory Response to Clinically Relevant Polymers by Manipulating the Host Response Using ITIM Domain-Containing Receptors

**DOI:** 10.3390/polym6102526

**Published:** 2014-09-29

**Authors:** Joshua B. Slee, Abigail J. Christian, Robert J. Levy, Stanley J. Stachelek

**Affiliations:** 1 Division of Cardiology, Department of Pediatrics, the Children“s Hospital of Philadelphia, Abramson Research Center, Suite 704, 3615 Civic Center Blvd., Philadelphia, PA 19104-4318, USA; 2 The Perelman School of Medicine, the University of Pennsylvania, Abramson Research Center, Suite 704, 3615 Civic Center Blvd., Philadelphia, PA 19104-4318, USA

**Keywords:** immunoreceptor tyrosine-based inhibitory motif (ITIM), SIRPα, CD47, PECAM-1, CD200R

## Abstract

Tissue contacting surfaces of medical devices initiate a host inflammatory response, characterized by adsorption of blood proteins and inflammatory cells triggering the release of cytokines, reactive oxygen species (ROS) and reactive nitrogen species (RNS), in an attempt to clear or isolate the foreign object from the body. This normal host response contributes to device-associated pathophysiology and addressing device biocompatibility remains an unmet need. Although widespread attempts have been made to render the device surfaces unreactive, the establishment of a completely bioinert coating has been untenable and demonstrates the need to develop strategies based upon the molecular mechanisms that define the interaction between host cells and synthetic surfaces. In this review, we discuss a family of transmembrane receptors, known as immunoreceptor tyrosine-based inhibitory motif (ITIM)-containing receptors, which show promise as potential targets to address aberrant biocompatibility. These receptors repress the immune response and ensure that the intensity of an immune response is appropriate for the stimuli. Particular emphasis will be placed on the known ITIM-containing receptor, Signal Regulatory Protein Alpha (SIRPhα), and its cognate ligand CD47. In addition, this review will discuss the potential of other ITIM-containing proteins as targets for addressing the aberrant biocompatibility of polymeric biomaterials.

## 1. Introduction

The host response to implanted, or extracorporeal, biomaterials is characterized by a nonspecific immune response to the biomaterial [1,2]. The pathophysiology associated with this biological process following medical device deployment represents a substantial healthcare burden [1]. Clinical issues have been reported as a result of acute and chronic inflammatory events directed at the synthetic surfaces that interact with host tissue. For example, the perfusion of large volumes of blood over polyvinyl chloride (PVC) blood conduits, used in such procedures as cardiopulmonary bypass or renal dialysis, elicits a systemic inflammatory response characterized by increased proinflammatory cytokines and neutrophil activation [3]. Detection of these chemokines and cytokines in the blood, during the procedure, correlates with poor clinical outcomes [4]. The response elicited by short-term exposure to foreign materials such as cardiopulmonary bypass and dialysis circuits represents an acute inflammatory reaction. The chronic inflammatory response has also been identified as having a deleterious role in long-term implanted devices. For example, the cracking of pacemaker lead insulation, which ultimately results in device failure, is mediated by the release of reactive oxygen species (ROS) from monocyte derived macrophages (MDMs) that respond to the polymeric insulation used in pacemaker leads [5,6]. In addition, addressing the issues surrounding aberrant host response to endovascular stents has been the subject of a great deal of resources expended by academic and industrial laboratories. Thus, attenuating biomaterial-induced inflammation by designing biomaterials that inhibit inflammation will address an unmet need in medicine, bioengineering, and biomaterial science. Central to this endeavor will be the achievement of a better understanding of the well-orchestrated molecular and cellular events that define the host reaction to synthetic surfaces.

A thorough characterization of the inflammatory response to biomaterials has been well documented by others [1,7], and is beyond the scope of this particular review. However, the cellular and molecular mechanisms of the inflammatory response will be briefly discussed herein to provide context for the necessity to establish a biomimetic surface that is based on a rational understanding of the cellular and molecular events that define biocompatibility. In general, the overall inflammatory response to biomaterials can be divided into an acute and a chronic phase that are defined by cell types, duration, and overall purpose. The initiation of the acute inflammatory response starts as a result of the tissue damage that is elicited when the medical device is implanted or, in the case of renal dialysis or cardiopulmonary bypass, when blood is perfused over the synthetic surfaces. This happens immediately after implant or blood material contact and is characterized by the adsorption of blood proteins such as albumin, antibodies, and fibrinogen onto the synthetic surface [8]. The exact profile of adsorbed proteins varies with the physical and chemical properties of the material used [9]. It is widely assumed that the presentation of the various plasma proteins on the material.’s surface, and not the material itself, creates a high affinity matrix for the subsequent attachment and activation of a range of inflammatory cells [10,11].

Similar to the process of wound healing, platelets are among the first cell type to respond to material implantation [12,13]. They respond by attaching to the provisional matrix on the material surface and then undergoing a series of morphological and physiological changes that lead to platelet aggregation and activation [12,13]. The activated platelets release an array of cytokines and chemokines that attract inflammatory cells such as macrophages and polymorphonuclear leukocytes (neutrophils, PMN), which in turn release more chemokines and cytokines that attract additional leukocytes to the site [12,13]. In short, the early inflammatory stages are defined by a regulated expression profile of molecular ligands that are released into the extracellular milieu with the overarching function of containing and removing the foreign stimuli.

**Figure 1:**
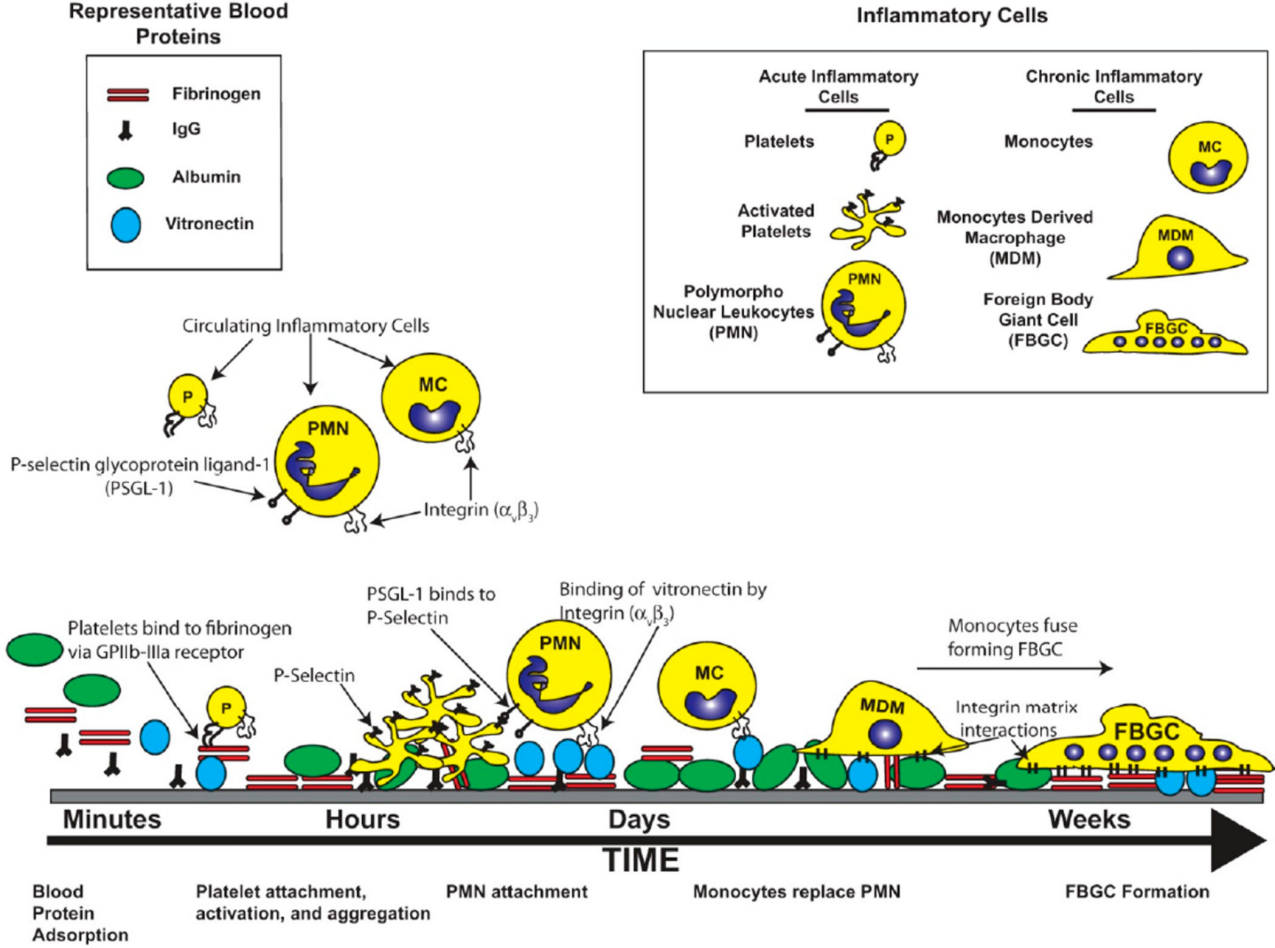
The Host Inflammatory Response to Biomaterials. The introduction of the synthetic surfaces, used in biomedical devices, initiates a host inflammatory response that involves the coordinated recruitment of pro-inflammatory cells and molecules. The initial stage of this process is the adsorption of blood proteins onto the material surface. This provides a high affinity matrix for the subsequent attachment of inflammatory cells. The population of the inflammatory cell type changes over time.

With respect to permanently implanted medical devices, the long term inflammation remains an important hurdle to achieving device efficacy. As the inflammatory response transitions from an acute to a chronic response, the cellular profile surrounding the implant changes [1,14]. During this time, the MDMs replace the PMN as the dominant cell type. The function of the MDM is generally to clear a foreign particle through phagocytosis. However, most medical devices are too large to be removed by phagocytosis. To that end, MDMs begin to remove the implanted material from the surrounding tissue through a two-step process of degrading the material and remodeling the surrounding extracellular matrix (ECM) from the surrounding host tissue [1,14]. The degradation of the implanted material is carried out by the release of hydrolytic enzymes and reactive oxygen and nitrogen species (ROS/RNS). Inflammatory cells produce a wide range of ROS/RNS through the activities of enzymes including NADPH oxidase, superoxide dismutase, and myeloperoxidase, as well as subsequent reactions with substrates such as the production of hydroxyl radicals from hydrogen peroxide and iron [15] or peroxynitrite from superoxide and nitric oxide [16]. It has been demonstrated that the reactions of ROS/RNS with a biomaterial can result in both reduction in composite polymer molecular weight and material degradation such as surface cracking of polyurethane-based pacemaker lead insulation [5,6] and structural damage to bioprosthetic heart valves [17]. This process is summarized schematically in Figure 1.

Depending upon the MDM phenotype, MDMs can facilitate tissue remodeling or fibrous tissue formation surrounding the biomaterial [18]. The phenotype of MDMs is generally characterized as M1 or M2. M1 are classically activated in response to stimuli such as pathogens and produce pro-inflammatory cytokines such as IL-1β whereas M2 are alternatively activated and have been shown to mediate a tissue remodeling response that can result in fibrous tissue formation [19]. MDMs with an M2 phenotype have been shown to release chemotactic factors that recruit cells such as myoblasts that are capable of forming an ECM network [19]. Over time, this ECM protein network will contract and remodel [1,14]. The process of remodeling the ECM involves the release of a family of enzymes known as matrix metalloproteinases (MMPs). MMPs are ubiquitously expressed endopeptidases, which collectively have the capacity of hydrolyze all components of the ECM [20,21]. MMPs are synthesized as inactive pro-enzymes which require activation by various proteinases and ROS/RNS to facilitate their transition to active enzymes [20,21]. Along with regulation of their enzymatic activity, MMPs are also regulated at the transcriptional and post-translation level [22]. Many inflammatory cytokines, such as tumor necrosis factor-alpha (TNF-α) and growth factors, such as epidermal growth factor (EGF) and transforming growth factor-beta (TGF-β) have been shown to upregulate MMP transcription [23]. Certain MMPs have also been shown to be regulated by the modulation of mRNA stability as is the case for MMP-3 [24]. Once activated, MMPs are then themselves regulated by general protease inhibitors and the family of tissue inhibitors of metalloproteinases (TIMPs) [25].

Although MMPs have been shown to degrade ECM components, they have also have been shown to be involved in the regulation of the inflammatory response. For example, several MMPs can cleave and activate TNF-α [26,27], interleukin-1 beta (IL-1β) [28], and TGF-β [29–31] increasing the inflammatory response. However, MMPs also possess anti-inflammatory capabilities. One of the more well-characterized examples of this is MMP-2-mediated cleavage of CC-chemokine ligand 7 (monocyte chemoattractant protein-3, MCP-3), generating a truncated version of MCP-3, which acts as a receptor antagonist inhibiting the inflammatory response [32]. It is clear that MMPs, which were previously thought only to be responsible for degradation of the ECM, have extensive involvement in modulating the immune response [21].

Device-associated inflammation remains a formidable obstacle in the development of various medical devices due to the pathophysiologies associated with long-term implants and blood-surface contacts. As detailed below, various therapeutic approaches to address the issues surrounding the device-associated inflammation have attempted to target the individual steps in the process. The strategies used can be generally placed into two categories. The first, bioinert strategies target events early in the inflammatory process, particularly minimizing the adsorption of proteins onto the material surface. Second, bioactive strategies aim to address specific events within the process. This review will focus primarily on the latter strategy.

## 2. Bioinert and Bioactive Surfaces

Bioinert surfaces are designed so that the chemical and topological features of the tissue contacting surfaces do not initiate an inflammatory reaction, and remain seemingly .“invisible.” to the immunosurveillance mechanisms that drive the host reaction to biomaterials. Specifically, these materials are largely designed to prevent the initial steps of the inflammatory process by preventing cell-material interactions [33]. Generally, the coatings used to make a bioinert surface tend to be hydrophilic as such surfaces do not enhance protein adsorption or cellular attachment [34], a problem which plagues hydrophobic coatings [35]. Various hydrophilic coatings have been shown to improve the biomaterial-tissue interaction, leading to long-term functionality of medical devices [34,36,37]. There is also evidence that altering surface topography, porosity, and structure can influence cell attachment to the biomaterial and the inflammatory response. For example, fibroblasts, epithelial cells, and endothelial cells have been shown to be influenced by alterations in surface topography [38–40]. Specifically related to the prevention of the inflammatory response, the use of porous implants in animal models have shown increased vascularization and decreased inflammation [41–44]. Similarly, microtemplating has been used to direct and organize cardiomyocyte bundles on sphere-templated porous materials used for cardiac tissue engineering [45]. These materials have also been documented to increase implant vascularization and prevent fibrosis associated with the foreign body reaction [45]. However, bioinert strategies have several limitations. First, it is unclear which types of surfaces confer a greater level of biocompatibility [33], and second no surface is free from the phenomena of protein adsorption (biofouling) [37], however ultra-low biofouling surfaces have been developed [46]. A variety of strategies have been employed to limit biofouling on biomaterials, however considerable success in preventing inflammation can be achieved using the ultra-low biofouling zwitterionic hydrogels prepared from carboxybetaine monomer and a carboxybetaine cross-linker [46]. These zwitterionic hydrogels exhibit ultra-low biofouling, lower COS-7 cell adhesions *in vitro* compared to poly(2-hydroxyethyl methacrylate) [47–49] and minimal foreign body capsulation around subcutaneous murine implants for up to three months [46]. Although early in their development, these bioinert compounds seem to have potential therapeutic applications.

Although there has been some success with bioinert strategies promoting biocompatibility, biomaterials as a field, has realized that masking a synthetic surface should not be the goal, but rather to promote healing and implant integration [50]. As bioinert surfaces aim to be invisible to immunosurveillance, a bioactive surface seeks to alter the biological response of inflammatory cells in a manner that preserves the efficacy of the medical device. Frequently, the therapeutic targets of bioactive coatings are components of the clotting cascade. The use of heparin on blood conduits has been suggested because of its anti-inflammatory and anti-proliferative capabilities [51,52]. For example, heparin-coated extracorporeal circuits, such as cardiopulmonary bypass circuits [53] have been shown to reduce the activation of coagulation, complement, and blood cells, thereby increasing their biocompatibility [54–59]. Encouraging results have also been obtained from co-immobilizing heparin and fibronectin on titanium implants, showing increased endothelialization and favorable blood biocompatibility [60,61]. Heparin-releasing hydrogels have also been shown to be successful in inhibiting the vascular smooth muscle cell (VSMC) proliferation around vascular stents, potentially increasing their long-term viability [62]. Although heparin has positive effects on acute inflammatory reactions associated with blood material interactions, these results have not translated into prevention of an inflammatory reaction when heparin-coated implants were evaluated in rats and heparin-coated coronary stents were evaluated in pigs [63–65].

The release or generation of nitric oxide from biomaterials has been widely explored with the overarching goal of preventing platelet activation and clotting on the material surface as well as preventing VSMC proliferation in the surrounding tissue [66]. Along with heparin and nitric oxide-based biocompatibility strategies, drug-eluting materials have also been developed and utilized in various applications. For example, antioxidant and paclitaxel-eluting vascular stents have been developed with the intention of promoting re-endothelialization of the stent (antioxidants) and inhibition of VSMC proliferation (paclitaxel) [67,68]. However, as with the majority of bioactive coatings on implanted materials, they are limited in certain aspects. For example, there is a finite amount of drug (or molecule) that can be delivered. This is a major limitation of most bioactive strategies, and many researchers are devising strategies to avoid this limitation. Another disadvantage of this type of strategy is the rate of release of the drug (or molecule). For example, the release rate of NO needs to be tightly regulated at a physiologic level to facilitate an anti-inflammatory effect, while accounting for the diffusion of NO to surrounding tissue [66]. Another limitation of all bioactive strategies is that the coating may become denatured or removed, due to biophysical interactions between the host tissue and the material. When the coating is altered in this manner, host proteins will replace the altered surface and may elicit an inflammatory response.

## 3. Immunoreceptor Tyrosine-Based Inhibitory Motif (ITIM)

Down regulation of the inflammatory response is maintained in part by a family of immune inhibitory receptors with a conserved amino acid motif (IVLS*X*Y*XX*LV; where *X* can be any amino acid) known as the immunoreceptor tyrosine-based inhibitory motif (ITIM) [69]. Ligand-induced signaling of ITIM receptors is mediated by tyrosine phosphorylation [69–71] and the downstream targets are often Src homology 2 (SH2) domain containing phosphatases, such as SHP-1 and SH2-containing inositol polyphosphate 5-phosphatase (SHIP) [69–71]. Once phosphorylated SHP-1 and SHIP activate distinct signaling pathways and elicit different changes in the inhibited cell [69]. Several reviews provide in depth discussion regarding their signaling mechanisms [72,73].

The ITIM family of proteins represents a large and diverse assembly of protein receptors of which some members lack an identified ligand. ITIM family members are essential negative regulators of the immune system ensuring a proportion response to inflammatory stimuli [69,70]. A delicate balance between activation and inhibition of the immune system is necessary to ensure the proper response. Failure of the inhibitory signal of ITIM receptors tips this balance towards an aberrant immune response and has been associated with various allergic and auto-immune diseases [74]. Below are several ITIM protein family members that may have the potential to regulate the inflammatory response. As detailed, some of these proteins are well characterized and may be easily translated to potential therapeutic technologies. In other cases, the underlying molecular physiology is not well understood and further investigations would yield both fundamental answers to the protein.’s function as well as increase the potential for developing platform technologies for addressing aberrant inflammatory events.

### 3.1. Signal Regulatory Protein Alpha (SIRPα)

SIRPα, an ITIM-containing transmembrane protein expressed in cells of myeloid origin, down-regulates the immune response through phospho-tyrosine signaling mechanisms [75]. CD47, the cognate ligand of SIRPα, is also a ubiquitously expressed transmembrane protein. SIRPα binding to the extracellular immunoglobulin (Ig) domain of CD47 is conserved and species specific, with sequence homology between mice and humans in this region differing by 38% [76]. Previous reports strongly suggest that CD47 functions as a .“marker of self.” by inhibiting immune cell interactions via SIRPα signaling [77–79]. CD47 is a member of the Ig superfamily with a single extracellular *N*-terminal IgV-like domain, 5 membrane spanning domains, and an intracellular alternatively spliced *C*-terminal domain [80]. CD47 is expressed on most cell types, including platelets [72,81,82] and a variety of other hematopoietic cells [83]. The removal of red blood cells from the circulation depends, in part, upon the level of CD47 expressed on their surface. As red blood cells age they express lower levels of CD47 and subsequently they are targeted for phagocytic degradation [84]. In biological systems, hematopoietic stem cells and leukemia cells evade phagocytosis partly by upregulating CD47 expression [85–87]. An emerging anti-cancer therapy which shows promising results is the use of antibodies which block CD47, allowing for the immune system to recognize cancerous cells which previously exhibit large amounts of CD47 [88]. In addition, myxoma viruses express a CD47 homologue presumably to evade phagocytosis [89]. In non-biological systems, CD47-SIRPα interactions have been shown to inhibit phagocytosis of opsonized microbeads [79]. Of all of the ITIM family of proteins, CD47 is likely the most well characterized for anti-inflammatory use on polymeric biomaterials.

**Figure 2:**
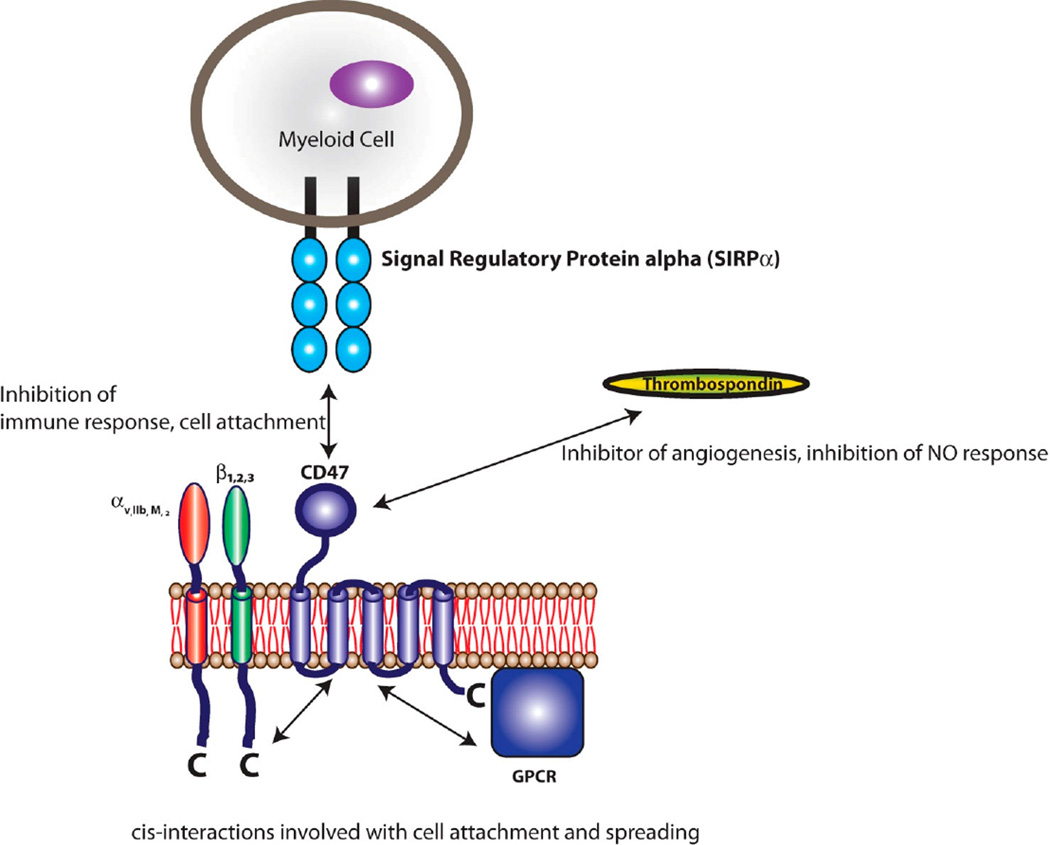
CD47 binding partners. CD47 is a transmembrane protein with an *N*-terminal extracellular IgV-like domain, 5 transmembrane domains, and a short *C*-terminal cytoplasmic tail. Well-characterized interactions with CD47 include: *cis* interactions with integrins, *trans* interactions with thrombospondin, and *trans* interactions with SIRPα.

**Figure 3:**
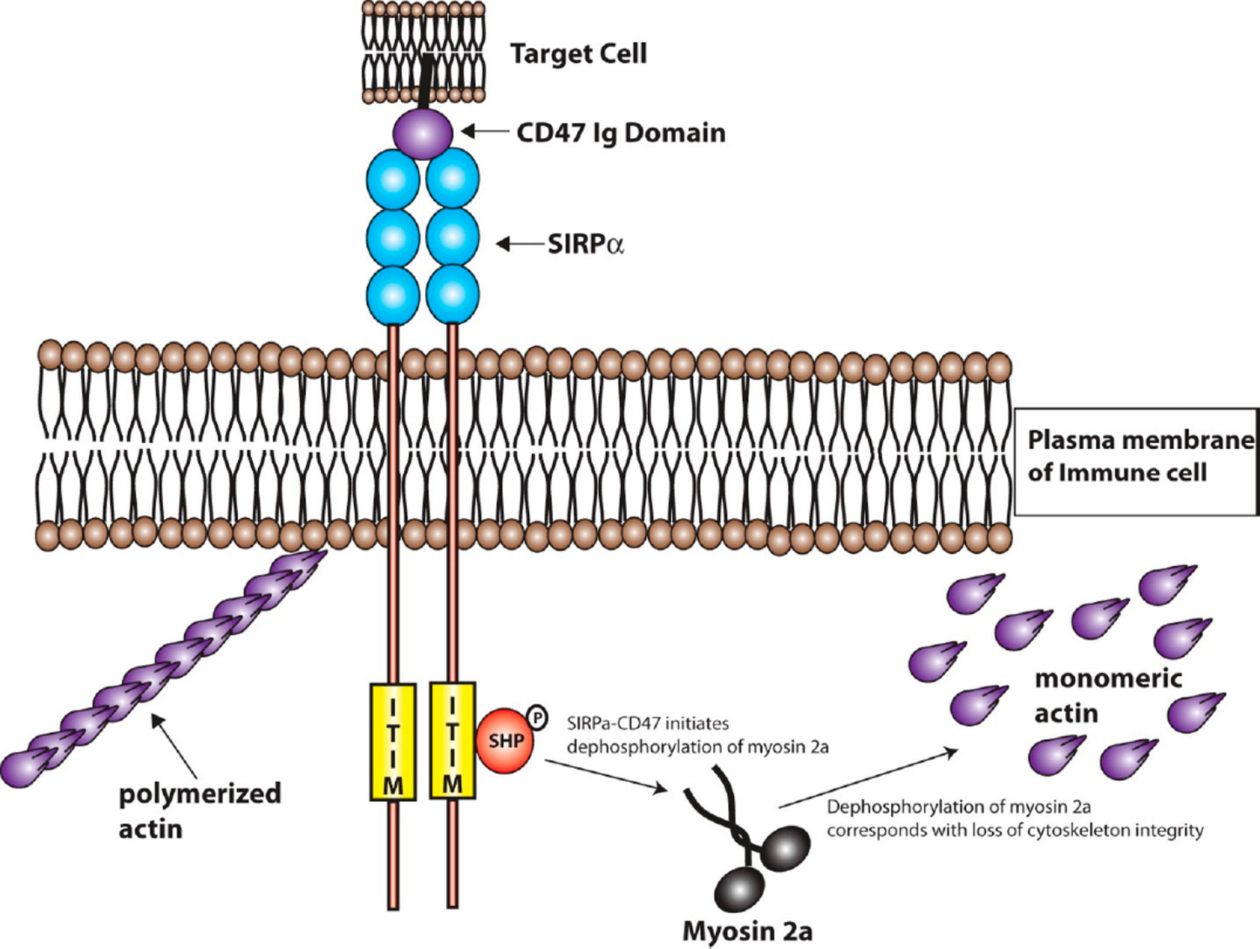
Signaling downstream of the SIRPα-CD47 Interaction. The interaction between CD47 and SIRPα activates the SIRPα ITIM domain via transphorylation events. This leads to the activation of SHP, the deactivation of myosin 2a, and initiates the depolymerization of actin within the immune cell. The depolymerization of actin is involved in inhibition of immune cell attachment and the phagocytosis of the CD47-bearing cell or biomaterial.

CD47 has been shown to interact in *cis* with integrins and in *trans* with thrombospondins [83]; but of particular importance to this review is the *trans* interaction among CD47 and SIRPα (Figure 2). The interaction between SIRPα and CD47 is involved in inhibition of cell growth, migration, and differentiation [72,73]. Specifically, SIRPα initiates a dephosphorylation cascade that ultimately targets myosin 2a and leads to the depolymerization of cytoskeletal actin [79]. The SIRPα-CD47 interaction is at its most basic sense anti-phagocytic, which can be in part, attributed to the deactivation of myosin 2a and the depolymerization of actin leading to decreased cell adhesion and decreased phagocytic activity (Figure 3) [79].

As a way of decreasing the inflammatory response to implantable devices and polymeric blood conduits, our lab and others have focused primarily on functionalizing surfaces with recombinant CD47. When recombinant CD47 is appended to polymeric surfaces, a stark reduction in inflammatory cell (human MDM THP-1 and human promyelocytic HL-60) attachment is observed compared to unmodified polymers using *in vitro* cell adhesion assays [81,90–92]. These inhibitory results translated well into the *ex vivo* blood perfusion model of the Chandler Loop and *in vivo* in rat sub-dermal implant models [81,90]. As alluded to previously, this inhibitory interaction between CD47-functionalized surfaces and inflammatory cells is mediated through the interaction of CD47 and SIRPα, demonstrated through the use of anti-SIRPα antibodies and *in vitro* cell adhesion assays [90]. Although the interaction between CD47 and SIRPα is often over-simplified to be anti-phagocytic or .“don.’t eat me.”, recent evidence from our lab indicates that this interaction involves much more than just .“don.’t eat me.”.

**Figure 4:**
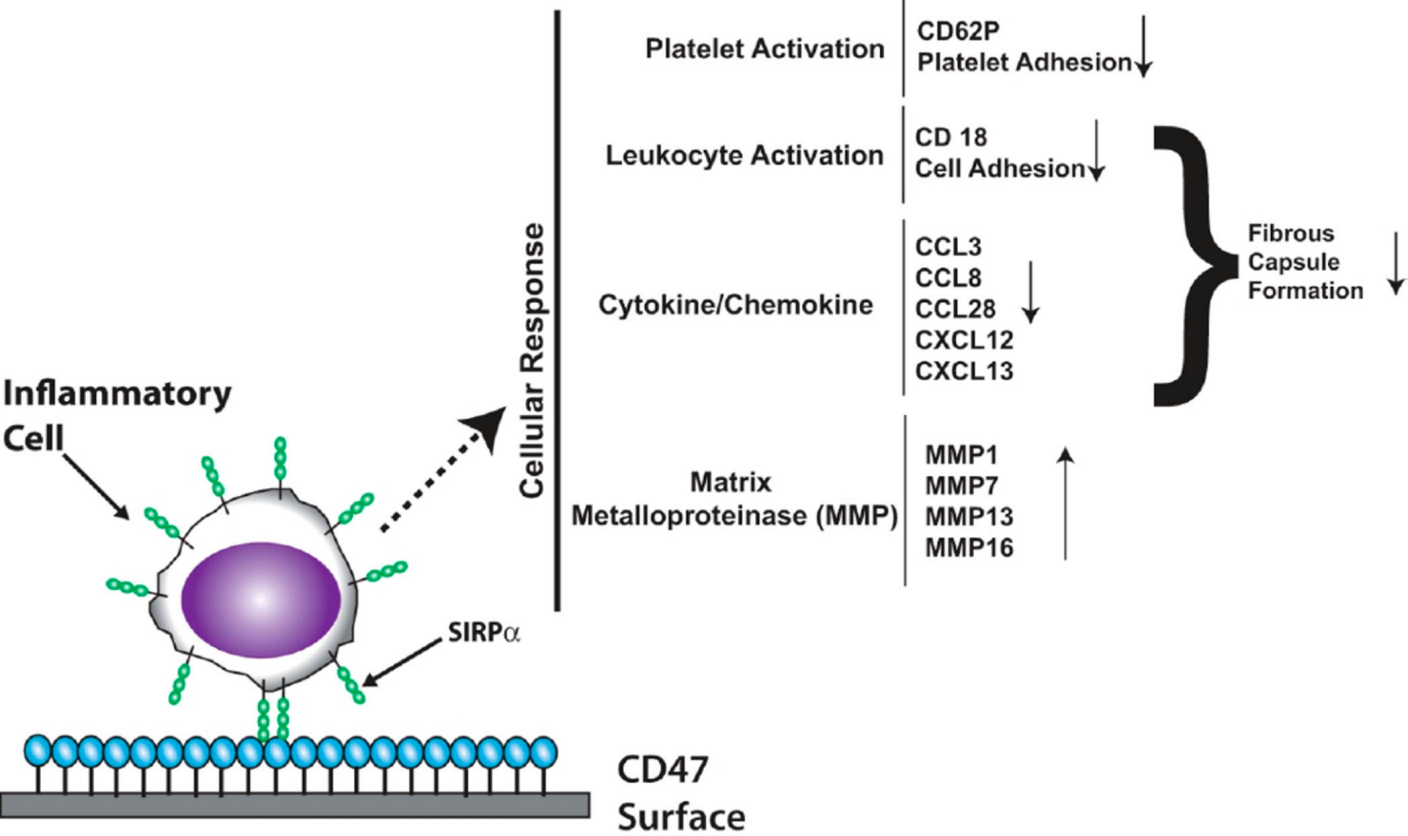
Genes regulated by the CD47-SIRPα interaction. Blood exposure to CD47-functionalized surfaces triggers signaling cascades that result in the down regulation of genes involved in platelet activation, leukocyte activation, and select cytokines/chemokines, and the up-regulation of *MMP* genes.

We have recently shown that the interaction of SIRPα with CD47-functionalized polymers elicits broadly defined intracellular signaling events [91]. CD47-mediated signaling regulates chemokine and cytokine transcription factors, increases MMP transcription, and reduces pro-inflammatory chemokines that are associated with poor clinical prognosis [91]. In addition, implicated in the signaling downstream of the SIRPα-CD47 interaction is the Janus Kinase/Signal Transducers and Activators of Transcription (JAK/STAT) pathway [91]. The exact role of the JAK/STAT pathway in biocompatibility regulated by SIRPα-CD47 remains unclear at this point; nevertheless it is evident that the pathway is involved. The signaling changes that are a result of blood exposure to CD47-functionalized surfaces are summarized in Figure 4.

A recent study [93] has shown that the peptide sequence of the Ig domain can confer roughly the same level of inhibition of phagocytosis of opsonized nanobeads as observed with recombinant CD47. As demonstrated previously by others [94–97], peptides have the following advantages over recombinant proteins: (1) Their ease of production contributes to lower manufacturing costs; (2) Peptides are more readily modified, thereby facilitating their chemical coupling to surfaces; (3) Peptides tend to be more biocompatible. Research into the use of CD47 peptide sequences could potentially provide a cost-effective bioactive surface for immunoengineering macroscale polymeric surfaces as a means to address host inflammatory response to implanted medical devices. Although the anti-phagocytic nature of CD47 Ig peptide seemingly depends on the interaction between SIRPα and CD47 [93], more recent literature suggests that the CD47 Ig peptide may not interact with SIRPα [98]. Regardless of the necessity of the interaction between SIRPα and the CD47 Ig peptide, the CD47 Ig peptide inhibits the phagocytosis of nanobeads by THP-1 cells [93], suggesting that this peptide should be investigated further.

Efforts by our laboratory and others have established the CD47-SIRPα signaling mechanisms as one of the more well-characterized ITIM proteins for use on biomaterials. However, as detailed below, other ITIM-expressing proteins may also be suitable for conferring biocompatibility upon synthetic surfaces used in medical devices.

### 3.2. Platelet Endothelial Cell Adhesion Molecule-1 (PECAM-1)

Platelet Endothelial Cell Adhesion Molecule-1 (PECAM-1), or CD31, is a member of the Ig superfamily containing two ITIM domains [99,100]. PECAM-1 is predominantly expressed on the lateral junctions of endothelial cells where it is involved in cell-cell junctions and is expressed at lower levels on platelets and leukocytes [101]. A variety of ligands for PECAM-1 have been established and include: homophilic interactions for cell adhesion among adjacent cells [102,103] and heterophilic interactions with integrins [104]. PECAM-1 also interacts with CD38 to regulate lymphocyte adhesion to endothelial cells [105,106].

The PECAM-1 cytoplasmic domain can transmit an immune inhibitory signal which is dependent upon its intact ITIM domain and the recruitment of SHP-2 and to a lesser extent SHP-1 [107,108]. PECAM-1 has also been implicated as a negative regulator of T cell receptor (TCR)-mediated signaling events [101,109], has established roles in leukocyte chemotaxis and transendothelial migration, and macrophage phagocytosis [101]. Although the mechanism remains unclear, healthy cell interaction with PECAM-1 leads to inhibition of phagocytosis, whereas this signal is interrupted in unhealthy cells resulting in their phagocytosis [110,111]. This suggests that modulation of PECAM-1 inhibitory signaling could be useful in preventing material-induced inflammatory events.

As discussed herein, the SIRPα-CD47 interaction and downstream signaling results in significant upregulation of MMPs. In support of the role of MMPs in the inhibition of the immune response, evidence suggests that PECAM-1 is regulated by MMP-dependent shedding which also involves caspase activity [112]. This shedding process results in the release of the soluble extracellular domain of PECAM-1 and a truncated transmembrane and cytoplasmic domain which preferentially recruits SHP-2 to enhance its signaling capabilities [112]. This presents the possibility of cross-talk between the two pathways, in which CD47 binds SIRPα, leading to an increase in MMPs. This increase in MMPs may themselves be involved in inhibition of the immune response, but they can also cleave the extracellular domain of PECAM-1. The remaining transmembrane and cytoplasmic fragment of PECAM-1 preferentially binds SHP-2 to strengthen its downstream signaling. To our knowledge no one has attempted to utilize PECAM-1 as an anti-inflammatory agent on biomaterials, but given its role in inhibiting T cell receptor activity, the potential exists to harness the anti-inflammatory nature of PECAM-1 for therapeutic benefit.

Although not investigated as an anti-inflammatory agent on biomaterials, important parallels in the literature can be identified. First, studies using *CD31* knockout mice demonstrate the requirement of PECAM-1 in modulating T-cell responses, because mice lacking *CD31* exhibited enhanced tumor and allograft rejection compared to wild-type mice [113]. The authors (Liang M. *et al*.) allude to the fact that this inhibition of T cell responses is presumably through the phosphorylation of the PECAM-1 ITIM domain, although the exact molecular mechanisms remain unclear [113]. Liang M. *et al*., in addition, postulated that selective expression of PECAM-1 might protect PECAM-1-expressing cells against cytotoxicity by effector T cells, rather directing them to PECAM-1 negative targets [113]. A similar proposal could be made for PECAM-1-functionalized biomaterials, which could be protected against cytotoxicity by effector T cells. Second, PECAM-1 has been used along with other cell adhesion molecules as a means to target drug nanocarriers to specific regions of the body by mimicking leukocyte rolling and extravasation [114–117]. In one particular example [114], PECAM-1 targeted nanocarriers and PECAM-1/Intracellular Adhesion Molecule-1 (ICAM-1) targeted nanocarriers were used to target endothelial cells *in vitro* and *in vivo*, facilitate endocytosis, and deliver a model therapeutic cargo in control and in inflammation-induced disease-like conditions. Although not directly related to the role of PECAM-1 as an ITIM-domain containing inhibitory receptor, these data demonstrate that PECAM-1-mediated cellular events can be manipulated for medicinal benefit.

### 3.3. CD200R

Another inhibitory immune receptor is CD200R which associates with its only established ligand, CD200, to transmit an immune inhibitory signal [118]. CD200 is widely distributed, having documented expression on thymocytes, T cells, B cells, dendritic cells, endothelium, hair cells, in neurons of the central nervous system, and cells in the retina and optic nerve [119,120]. The current model in the literature is that CD200 lacks intracellular signaling due to its short cytoplasmic tail and its only established purpose is to bind and activate CD200R [121]. The expression of CD200R is restricted to macrophages, granulocytes, dendritic cells, T cells, B cells, and natural killer cells [122–124]. The CD200–CD200R interaction has been well-characterized to inhibit the activation of myeloid cells as a way of exerting its immune inhibitory effects [125–128]. Although often grouped with immune inhibitory receptors bearing cytoplasmic ITIM domains, CD200R lacks a classical ITIM domain but rather has three tyrosine residues that may be important for its inhibitory functions [74]. In humans, the most membrane distal tyrosine residue is located within a phosphotyrosine-binding (PTB) domain recognition motif (NPXY) which has been shown to facilitate the majority of the intracellular signaling [129,130]. Interestingly, human, mouse, rat, and cow CD200R lack a classical ITIM, while chicken CD200R contains a classical ITIM sequence (NVIYNSV) [123,125,131], suggesting that mammalian CD200R may have evolved from an ITIM-bearing receptor [132].

CD200 binding to CD200R triggers rapid tyrosine phosphorylation events mediated through the distal most PTB domain of CD200R, leading to the phosphorylation of downstream of tyrosine kinase (Dok) 1 and Dok2 [128,130,132]. Subsequently, Dok2 is involved in recruiting Ras GTPase-activating protein (RasGAP) and SHIP to facilitate downstream signaling. RasGAP can directly inhibit extracellular signal-related kinase (ERK) activity, while SHIP is presumably involved in inhibiting p38 mitogen-active protein kinase (MAPK) and Jun amino-terminal kinase (JNK), because their activity is independent of RasGAP. Although the definitive signaling pathways are still under investigation CD200R ligand binding clearly decreases the activity of the above MAPKs [130]. Initially, Dok1 was thought to play a complementary role to Dok2 to inhibiting the immune response, until recently it was suggested that Dok1 and Dok2 have opposing roles in regulating the immune response. Dok1 which is activated by a separate phosphotyrosine residue on CD200R, complexes with CT10 sarcoma oncogene cellular homologue-like (CrkL) to inhibit Dok2 phosphorylation. This facilitates a negative feedback loop preventing the activation of RasGAP, thereby preventing Dok2-mediating immune cell inhibition [133].

In many aspects the CD200-CD200R system is most similar to the CD47-SIRPα system. Similar to CD47, CD200 is overexpressed in many cancers as a mechanism to avoid immunosurveillance detection by CD200R-containing leukocytes [134–138]. In addition, CD200 homologs have been detected in many parasites, bacteria, and viruses as a way to prevent immune recognition of infected cells [139–144]. Age and/or disease-related decreases in CD200 has been shown to be involved in chronic inflammation, particularly in the brain where it may play a role in the development of multiple neurodegenerative diseases [121]. This observation coupled to the notion that CD200 is considered an .“off.” signal for many immune cells presents the possibility that CD200 could be used as a therapeutic intervention whenever inhibition of immune cell activity would be instrumental in facilitating a positive physiologic outcome. Although research is limited on using CD200 as a therapeutic, some laboratories have focused on using CD200 blocking antibodies to prolong the survival of renal and cardiac allografts [145], decrease inflammation associated with arthritis [146], various autoimmune diseases [147], and a subset of cancers involving upregulated CD200 [148]. A soluble form of the CD200 protein has been shown to reduce microglia markers of neuroinflammation when injected into the hippocampus of aged or lipopolysaccharide-treated rats suggesting that CD200 can be used as an anti-inflammatory agent [121,149]. A study that was just published investigated the potential of using CD200 as an anti-inflammatory agent on biomaterials [150]. The authors generated biotinylated CD200 and immobilized it onto streptavidin-coated polystyrene surfaces as a model biomaterial. Their *in vitro* analysis demonstrated decreased macrophage inflammatory activation and decreased macrophage secretion of TNFα and interleukin-6 (IL-6) compared to control and streptavidin-coated polystyrene surfaces. To demonstrate that the decreased macrophage activation was specifically due to their interaction with CD200, blocking antibodies against CD200 were used, which resulted in increased inflammatory responses compared to control surfaces. Given the encouraging nature of their *in vitro* data, the authors also examined the effects of CD200 on polystyrene microbeads injected subcutaneously into mice. Tissue surrounding the injected microbeads was analyzed 24 h post implant for makers of inflammation. The CD200-coated microbeads exhibited significantly less infiltrated cells and ROS compared to control and streptavidin-coated beads, supporting their *in vitro* analyses [150]. To our knowledge this is the first article of its kind to conclusively document the anti-inflammatory capacity of CD200 on biomaterials and support the notion that modulating the immune response to biomaterials will likely provide the best route to biocompatibility.

## 4. ITIM Receptors and the Adaptive Immune Response: Implications for Biomaterials

Although the response to biomaterial surfaces is largely dictated by innate immune mechanisms and the physical-chemical surface properties of the individual material, there is a growing interest into the adaptive immune response to clinically relevant biomaterials. Briefly, the adaptive immune response is largely defined by the response of T and B lymphocytes to specific antigens. Many biomaterials, such as self-assembling peptide nanofibers, decellularized tissue, and multilaminar vesicles [151] are able to elicit an antibody response. As interest in the use of certain biomaterials for vaccine development is growing, the ability of a biomaterial to elicit an immune response may be desired. However, for many applications the establishment of a bioactive biomimetic surface is desirable.

As shown in Table 1, T and B cells express several ITIM family members. B cell activation and proliferation are controlled in part by several ITIM-containing molecules including FcγRIIB, CD22, CD72, and PECAM-1. As such, these proteins could be potential therapeutic targets in preventing allograft rejection. For example, Fibrinogen-like protein 2 (FGL2) binding to FcγRIIB results in immune suppression [152]. Thus, a FGL2 functionalized surface may confer immunotolerance to those implanted materials where immunogenicity is a concern.

Cytotoxic T lymphocyte associated antigen-4 (CTLA-4) is one of the few ITIM-containing receptors, related to adaptive immunity, that has been studied within the context of biomaterials research [153]. A study looking at dendritic cell (DC) maturation as a function of the surface of self-assembled monolayers (SAMs) presenting various end group chemistries (–OH, –COOH, –CH_3_, or NH_2_), showed that –CH_3_ modified SAMS attained the least level of DC maturity, which coincides with an increased immune response. The study also observed an increased level of CTLA-4 on T cells following –CH_3_ SAM exposure. These observations may begin to define CTLA-4 as a potential mediator for inhibiting the T-cell responses to biomaterials.

Programmed death-1 (PD-1), is expressed in activated T cell, B cells, mast cells and monocytes. When bound to its ligands, PD-L1 and PD-L2, PD-1 delivers an inhibitory signal that has been shown to inhibit the immune response. Targeting PD-1 has been the focus of several laboratories that investigate graft *vs*. host disease (GVHD). As such, research into PD-1 as a therapeutic target to mask implanted synthetic surfaces may be a viable strategy. Research into the identification and characterization of ITIM-containing proteins that regulate the adaptive immune response to biomaterials is still in its early stages. However, it remains an important unmet need in pharmacology and organ transplantation as knowledge regarding ITIMs in this capacity can be used to improve vaccine delivery systems as well as direct therapeutic strategies to mitigate the GVHD in allograft and xenograft transplanted tissue. Fortunately, laboratories [151,154,155] have increasingly began to investigate the effect of biomaterials upon T-cell and B-cell function and advancements in the field seem likely.

## 5. Limitation of ITIM-Based Therapeutics

The major limitation of functionalizing biomaterials with ITIM receptor ligands is that they are still fairly uncharacterized and a relatively understudied area of the immune system. Thus far, the interaction between SIRPα and CD47 is the only characterized ITIM interaction that has been applied as a functionalized coating on implantable polymers and our understanding of this system is still in its infancy. However, the same limitations for other bioactive strategies apply to the use of ITIM domains as well. First, no surface modification strategy characterized thus far has been shown to completely prevent protein adsorption, a common problem associated with blood contacting surfaces and an initiating step of the material induced inflammation. Therefore, protein adsorption will still likely be an issue with ITIM-based strategies. Second, over time the coating can become denatured or removed due to the interaction between host tissue and the material. Once the coating is removed, the material will elicit an inflammatory response. Third, the attachment method used to functionalize the material surface can hinder the bioefficiency of the implanted material. Ultimately, the bioactive immune inhibitory strategy must preserve the efficacy of the device itself while attenuating the inflammatory response. These limitations will need to be addressed universally for any bioactive coating to be effective.

A limiting factor to targeting ITIM-bearing receptors using their cognate ligands is that they are limited to the inhibiting the cells which express that particular ITIM. This does not seem to constrain the use of the CD47-SIRPα interaction, because SIRPα is expressed on most cells of myeloid origin. However, if widespread immune cell expression is not seen with a particular ITIM, bifunctionalization with multiple ITIM-bearing receptor ligands or other bioactive strategies may be a viable strategy. A limitation of using any recombinant protein as an anti-inflammatory strategy is the cost associated with their production and the amount needed to functionalize surfaces. For example, modification of large scale surfaces such as cardiopulmonary bypass and hemodialysis tubing requires a significant amount of recombinant protein. Therefore, it might be more advantageous to explore the use of peptides in place of recombinant proteins. As previously mentioned, compared to recombinant proteins, peptides are generally easier to manufacture thereby lowering costs, peptides are more readily modifiable to facilitate surface coupling, and tend to be more biocompatible [94–97]. Thus, synthetic peptides, corresponding to ligands of ITIM expressing proteins, may be a cost-effective alternative to recombinant proteins in preventing material-induced inflammation on macroscale surfaces.

## 6. Conclusions

Aberrant biocompatibility of implanted biomaterials and devices are a significant burden to the healthcare system and account for a large proportion of post-surgical clinical complications [1]. Targeting the body.’s natural mechanisms of inhibiting the immune response is a logical way to combat inflammation caused by implantable materials. This can be accomplished specifically through utilizing the ITIM family and related immune inhibitory receptors, which are involved in attenuating the inflammatory response. Given that these inhibitory receptors are expressed on immune cells makes them attractive targets for drug design or functionalization on implantable devices. Herein, we discussed the potential use of a select few immune inhibitory receptors some bearing classical ITIMs and others with alternative signaling mechanisms in attenuating the inflammatory response. We detailed the potential uses for recombinant CD47, the ligand for the ITIM receptor SIRPα, that we believe to be the best suited for promoting long-term biocompatibility of implanted medical devices. The further we expand our understanding of the role that ITIM proteins have in fine tuning the immune response, the better suited we will be to utilize ITIM proteins to increase medical device biocompatibility.

## Figures and Tables

**Table 1:** Representative immunoreceptor tyrosine-based inhibitory motif (ITIM) receptors expressed on T and B cells.

Receptor	T or B Cell Distribution	Ligand
FcγRIIB	B	IgG
CTLA-4	T	CD80, CD86
PD-1	T	PD-1 ligand 1 and 2
CD72	B	Unknown
CD22	B	Sialic Acid
CD66a	T,B	CD66, CD62E
